# Is sympatric speciation in nature only possible with micro-parapatry? A new case study of glacial lake cyprinid fishes

**DOI:** 10.1093/nsr/nwad006

**Published:** 2023-02-02

**Authors:** Christopher H Martin

**Affiliations:** Department of Integrative Biology, University of California, Berkeley, USA; Museum of Vertebrate Zoology, University of California, Berkeley, USA

Although numerous theoretical models of the feasibility and necessary conditions for sympatric speciation abound in the literature, clear-cut examples in nature remain elusive. And, as always in speciation research, this statement strongly depends on your definition of sympatric speciation [1–3]. If using the biogeographic definition of sympatric speciation as divergence between individuals in a population within ‘cruising range’ of each other [4], then empirical case studies are plentiful [5–8]. We have previously referred to this as the ‘easy’ process of sympatric divergence because secondary gene flow is permissible under this definition [1]. In this issue, Sun et al. refer to this as micro-parapatric speciation, correctly acknowledging that some spatial structure within a lake may contribute to assortative mating and facilitate sympatric divergence [9]. This process is ‘easy’ because any form of pre-existing reproductive isolation accelerates the speciation process and expands the parameter conditions under which a population can diverge.

In contrast, the truly elusive beast, that original sought-after Lernaean Hydra, is empirical evidence in nature for sympatric speciation under the population genetic definition [1]. This requires complete panmixia at the initiation of the speciation process and no contributions to reproductive isolation due to secondary gene flow (although gene flow that does not affect reproductive isolation would be permissible since it would not have facilitated divergence). This strict definition also explains why empirical examples of ‘hard’ sympatric speciation are so rare. Demonstrating sympatric speciation under the population genetic definition requires not only fulfilling Coyne and Orr's original strict criteria of reproductively isolated sympatric sister species with no historical periods of allopatric isolation, but now in the genomic age it also requires demonstrating that any secondary gene flow during the process of sympatric divergence played no role in contributing to reproductive isolation [10]. It is also worth noting that every single putative case study of sympatric speciation investigated at the genomic level so far shows evidence of secondary gene flow [1], so it is a tall order to either show that these are false positives or demonstrate—preferably with functional tests—that none of the introgressed alleles contributed to assortative mating or ecological divergence.

Sun *et al*. correctly and humbly conclude that they have not found the elusive Hydra, but instead report a new case of micro-parapatric speciation in a sister species pair of glacial lake fishes. Sun *et al*. stick to the original population genetic definition of allopatry as zero gene flow between species and the population genetic definition of sympatric speciation, so they are able to reject both the allopatric and sympatric models of divergence. Rather than argue for another case of sympatric speciation, they acknowledge that this case study meets the biogeographic criteria of sympatric speciation within cruising range and, instead, humbly conclude that micro-parapatry is the best model to explain speciation in this system. They constructed a new high-quality *de novo* genome assembly and resequenced 52 resequenced genomes of the two species to infer their demographic history and patterns of genetic differentiation. The authors find many large regions of differentiation between these species which are primarily enriched for olfactory receptor genes and inferred to play a role in the speciation process. This is strikingly consistent with another case study of micro-parapatric speciation in tiny Lake Ejagham in Cameroon, in which secondary gene flow of olfactory receptor genes may have triggered rapid sympatric divergence of cichlids after approximately 8 000 years without divergence despite continuous gene flow into the lake [11].

This case study is critically important because cypriniform fishes have radiated in sympatry so rarely across the entire planet, perhaps only three or four times. This includes the large radiation of mostly piscivorous barbs in Ethiopia's Lake Tana [12] and the once incredibly diverse radiation of morphologically disparate barbs in Lake Lanao, Philippines, in which nearly all species are now extinct [13]. In the age of the Anthropocene and the ongoing sixth global mass extinction of biodiversity, it is urgent to study these rare sympatric adaptive radiations of cypriniforms to understand what processes could drive sympatric divergence in these toothless fishes.

Sun *et al*. also ran demographic simulations of selection on the introgressed regions between species and argue that the large size of regions of genetic differentiation between species they observed rule out the possibility of sympatric speciation because large introgression tracts should have been quickly broken down by recombination since the start of speciation. However, they also report reduced rates of recombination in the most differentiated regions between species, suggesting that sorting of ancestral polymorphism or secondary gene flow of structural rearrangements could also play a role in species divergence [14]. Inference of the sizes of introgressed tracts and genomic analyses of structural variants would be warranted in future studies [15,16]. Nonetheless, the overall conclusion that large regions of genetic differentiation between species should give us pause for thought before concluding that speciation occurred in sympatry is sound. For example, inversions may result in the physical linkage of assortative mating and ecological loci and prevent their disassociation due to recombination, as in sunflowers [17], or predate the speciation event, as in *Drosophila pseudoobscura* and *D. persimilis* [18].

Importantly, Sun *et al*. conclude that many—I would suggest perhaps even all—reported cases of sympatric speciation are instead likely to be examples of micro-parapatric speciation when using the strict population genetic definition of sympatric speciation. In nearly every known example of sympatric speciation reported in the literature there is a mechanism of ‘mate-where-you-eat’ assortative mating, i.e. micro-parapatry [1]. This mechanism is extraordinarily important and pervasive throughout case studies of speciation because it results in the automatic linkage of ecotype and assortative mating [19], comparable to the geographical linkage of ecology and assortative mating in allopatric speciation [20]. Can sympatric speciation occur in nature without the benefit of a mate-where-you-eat mechanism? Are most examples of micro-parapatric speciation (‘easy’ sympatric speciation) also linked to olfactory mating preferences? This important case study adds more fuel to these emerging questions in speciation research.
